# Outbreak of Campylobacteriosis Associated with a Long-Distance Obstacle Adventure Race — Nevada, October 2012

**Published:** 2014-05-02

**Authors:** Mariah Zeigler, Chad Claar, Daviesha Rice, Jack Davis, Tammy Frazier, Alex Turner, Corinna Kelley, Jonathan Capps, Andrea Kent, Valerie Hubbard, Christiana Ritenour, Cristina Tuscano, Zuwen Qiu-Shultz, Collette Fitzgerald Leaumont

**Affiliations:** 1 Nellis Air Force Base Public Health Flight, Nellis Air Force Base, Nevada; 2 Southern Nevada Health District, Las Vegas, Nevada; 3 Division of Foodborne, Waterborne, and Environmental Diseases, National Center for Emerging and Zoonotic Infectious Diseases, CDC

On October 12, 2012, the Nellis Air Force Base Public Health Flight (Nellis Public Health), near Las Vegas, Nevada, was notified by the Mike O’Callaghan Federal Medical Center (MOFMC) emergency department (ED) of three active-duty military patients who went to the ED during October 10–12 with fever, vomiting, and hemorrhagic diarrhea. Initial interviews by clinical staff members indicated that all three patients had participated October 6–7 in a long-distance obstacle adventure race on a cattle ranch in Beatty, Nevada, in which competitors frequently fell face first into mud or had their heads submerged in surface water. An investigation by Nellis Public Health, coordinated with local and state health officials, identified 22 cases (18 probable and four confirmed) of *Campylobacter coli* infection among active-duty service members and civilians. A case-control study using data provided by patients and healthy persons who also had participated in the race showed a statistically significant association between inadvertent swallowing of muddy surface water during the race and *Campylobacter* infection (odds ratio = 19.4; p<0.001). Public health agencies and adventure race organizers should consider informing race attendees of the hazards of inadvertent ingestion of surface water.

*Campylobacter* is one of the most common causes of diarrheal illness in the United States. Most persons who become ill with campylobacteriosis get diarrhea, cramping, abdominal pain, and fever within 2–5 days after exposure to the organism. The diarrhea can be bloody and can be accompanied by nausea and vomiting. The illness typically lasts about 1 week. Most cases occur as isolated, sporadic events and are usually associated with eating raw or undercooked poultry or from cross-contamination of other foods by these items (1).

## Initial Epidemiologic Investigation

Because of the three cases of hemorrhagic diarrhea and the suspected source of infection reported to Nellis Public Health by ED staff members on October 12, definitions were developed to identify additional cases. A probable case was defined as diarrhea (three or more loose stools in a 24-hour period), any episode of bloody diarrhea, or a combination of other gastrointestinal illness symptoms (e.g., abdominal cramps, nausea, or vomiting) in a person who participated in the obstacle adventure race during October 6–7. A confirmed case was defined as a probable case in a patient who also had laboratory isolation of *Campylobacter* from a stool specimen.

An additional 19 patients, including both military and civilian personnel, were identified through active reporting by clinical staff members throughout MOFMC, a retrospective review of ED logs from October 6–16, and announcements to the Nellis community that encouraged self-identification. These efforts resulted in the identification of a total of 18 probable and four confirmed cases of illness. The investigation was limited to the population of the Nellis community, primarily because of the short incubation period for *Campylobacter*, the time lags between the event, symptom onset, and investigative findings, and the lack of additional cases reported to the Southern Nevada Health District by civilian health-care providers.

Among the 22 patients, the mean time from exposure to illness was 3.3 days (range = 1–9 days) (Figure). The most common symptoms were diarrhea (18 of 19 patients), cramps (14 of 18 patients), fever (10 of 18 patients), and nausea (10 of 17 patients) (Table 1). Twenty of the 22 patients sought medical care, and two reported their illness directly to Nellis Public Health without seeking care. One person with chronic gastrointestinal illness was hospitalized and treated with supportive care and intravenous antibiotics. All 22 patients made a full recovery.

To obtain information about the outbreak source, a 72-hour food and drinking water history questionnaire, which included questions on surface water exposure, was used to interview the ill persons. An analysis of the questionnaire data indicated that muddy surface water was a possible source of infection.

## Case-Control Study

A case-control study was conducted to identify the source of infection. Twenty-four healthy controls consisting of both military personnel and civilians who had been race participants were identified through contact investigation of the 22 case-patients. Nellis Public Health developed a new questionnaire for this investigation and administered it by telephone. The questionnaire evaluated the 22 case-patients and 24 controls with regard to their water consumption, food consumption, and environmental water exposure during the October 6–7 obstacle race.

Analysis of the case-control study identified a statistically significant association with “inadvertent swallowing of muddy water while competing” and *Campylobacter* infection (odds ratio = 19.4; p<0.001) (Table 2). No significant association (p<0.05) was found with drinking water or eating food provided by race organizers, full body submersion in surface water, or getting surface water or mud in the eyes or mouth.

## Laboratory Testing

Nellis Public Health requested stool specimens from all 22 patients and recommended cultures for *Shigella, Campylobacter, Salmonella,* and *Escherichia coli* 0157:H7, plus testing for Shiga toxin and a search for ova and parasites. Initially, four stool specimens were obtained and each tested negative for all organisms, including *Campylobacter*. Persistence in obtaining seven additional stool specimens resulted in four laboratory-confirmed cases positive for *Campylobacter* by growth on selective media, oxidase testing, and Gram stain at the MOFMC laboratory. These four isolates were identified as hippurate-negative *Campylobacter* (not *Campylobacter jejuni*) by the Southern Nevada Health District Public Health Laboratory and further identified as *Campylobacter coli* by CDC.

Further characterization of the four *C. coli* isolates by pulsed-field gel electrophoresis using *SmaI* and *KpnI*, multilocus sequence typing, and antimicrobial susceptibility testing at CDC, identified all four as the same strain. This *C. coli* outbreak strain was pansusceptible to the antimicrobials tested on the CDC national antimicrobial resistance monitoring system panel, and was assigned PulseNet patterns DBBS16.0134/DBBK02.0272 and sequence type (ST) 6159. All specimens tested for *E. coli* 0157:H7, *Salmonella, Shigella*, Shiga toxin, and ova and parasite testing were negative.

## Public Health Action

Because commercial obstacle adventure races often are marketed to military personnel, Nellis Public Health provided educational outreach to the base population regarding the risk for disease when competing in such events. Emphasis was placed on the importance of hand washing and avoidance of exposure (especially ingestion) to contaminated surface water to prevent disease. This investigation also highlighted the importance of outbreak investigators continuing stool specimen collection, culture, and serial testing, even after initial results are negative.

### Discussion

Inadvertent ingestion of muddy surface water contaminated with cattle or swine feces during a long-distance obstacle adventure course competition likely resulted in an outbreak of campylobacteriosis in 22 participants. Four of the 22 had laboratory-confirmed infections with *Campylobacter coli*.

High-intensity and competitive muddy obstacle adventure course races have surged in popularity across the United States, drawing an estimated 1.5 million participants in 2012 (2). These military-style adventure races attract high numbers of active-duty military personnel, along with young, active, extremely fit civilians. Persons typically are advised of the risks of participating and required to sign a liability waiver. Races are commonly held on farmlands where animal feces increase the risk for zoonotic disease transmission. Primary and emergency care providers, as well as public health professionals, should be aware that obstacle adventure race events could pose a heightened risk for outbreaks from inadvertent ingestion of contaminated water or mud and might consider outreach to educate participants on the health risks from oral contact with contaminated surface water or mud.

Documented common-source outbreaks of campylobacteriosis (especially those caused by *C. coli*) are rare, but have been previously attributed to contact with nonchlorinated water contaminated with the feces of cattle, poultry, and swine (3). *Campylobacter* is an important cause of acute zoonotic bacterial diarrhea across all age groups. An estimated 5%–14% of diarrhea cases worldwide are attributed to this organism, and approximately 2.4 million human cases of campylobacteriosis occur annually in the United States (4).

Participation in obstacle adventure races is relatively common among men and women of the U.S. military. These events typically are held in rural areas and often include man-made slurry fields (a mixture of soil or clay and water) as race “challenges.” In areas commonly frequented by animals (5), topsoil used in the creation of slurry fields can be contaminated with feces from domestic fowl (6) or ruminants (7) or wild animals. Competitors who run or ride through such areas might unintentionally swallow sufficient numbers of organisms to cause clinical disease. Fewer than 500 *Campylobacter* organisms are needed to cause illness (1). The race described in this report was held on a cattle ranch, and participants reported seeing cattle and swine on or near the course on race day. Obstacle adventure race planners should consider building slurry field challenges where animal fecal contamination is not likely.

Although contaminated food and drinking water are more common sources of *Campylobacter* outbreaks, previous outbreaks have been associated with unintentional ingestion of contaminated mud or muddy water. Campylobacteriosis outbreaks were associated with two bicycle races in Norway in the 1990s, in which unintentional ingestion of dirty water splashing from bicycle wheels was implicated (8). Similarly, ingestion of mud was found the most likely cause of *Campylobacter* outbreaks during mountain bike races in Wales in 2008 (9) and in British Columbia in 2010 (10).

Warning participants in outdoor sporting events who might be exposed to fecally contaminated water or slurry that potentially serious diarrheal disease can result if ingested, even inadvertently, could reduce exposures to these pathogens. Event organizers should consider including the risk for waterborne outbreaks in their participant waivers and advise participants to avoid drinking or swallowing unsafe water. Participants also need to be encouraged to seek appropriate medical care for postcompetition diarrhea, especially bloody diarrhea, and to inform medical personnel of their exposure. In addition, health-care providers need to be aware of the association between these adventure races and the risk for exposure to *Campylobacter* or other pathogens via contaminated water, mud, or slurry so that appropriate diagnostic testing and treatment can be provided to ill participants.

What is already known on this topic?

*Campylobacter* is an important cause of acute zoonotic bacterial enteric disease worldwide. The most common cause of campylobacteriosis in humans is *Campylobacter jejuni*, with *Campylobacter coli* less common. Livestock, including cattle and swine, are important reservoirs for human infection with *C. coli*. Multiple outbreaks have been linked to contaminated surface water.

What is added by this report?

In 2012 a total of 22 cases of acute diarrheal disease attributed to *C. coli* were identified among participants in a long-distance obstacle adventure race in Beatty, Nevada. Eleven stool specimens were collected for culture, and four were positive for *C. coli*. This investigation established an association between inadvertent swallowing of muddy surface water and *C. coli* infection. In addition, the investigation demonstrated the potential need for ongoing collection of stool specimens for culture during a food or waterborne outbreak to identify the causative agent and implement public health preventive measures.

What are the implications for public health practice?

Participation in adventure races combining mud and obstacles has become popular with extremely fit members of the general public, including military personnel. The races often take place on farmland exposing participants to numerous zoonotic pathogens. This outbreak highlights *C. coli* as a cause of diarrhea associated with such exposures and the importance of informing participants and race organizers regarding these hazards.

## Figures and Tables

**FIGURE:**
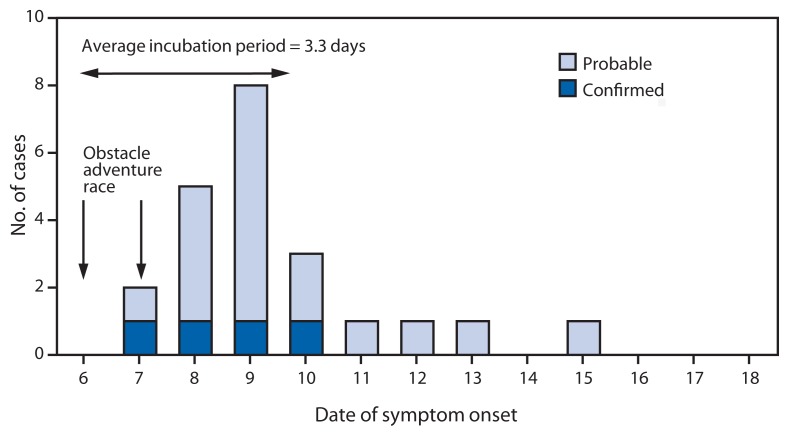
Number of probable and confirmed cases of *Campylobacter coli infection* among participants in a long-distance obstacle adventure race, by date of symptom onset — Nevada, October 2012

**TABLE 1:** Number of persons (N = 22) with signs and symptoms of confirmed or probable *Campylobacter coli* infection after participating in a long-distance obstacle adventure race — Nevada, October 2012

Sign/Symptom	No.*	(%*)
Diarrhea	18 of 19	(95)
Cramps	14 of 19	(74)
Fever	10 of 18	(56)
Nausea	10 of 18	(56)
Vomiting	9 of 17	(53)
Watery diarrhea	7 of 10	(70)
Bloody diarrhea	6 of 10	(60)
Influenza-like illness	6 of 10	(60)
Mucus-like diarrhea	3 of 10	(30)
Chills	3 of 7	(43)

* Denominator values varied as a result of nonreporting by some participants.

**TABLE 2:** Comparison of case-patients with *Campylobacter coli* infection and control subjects among participants in a long-distance obstacle adventure race, by food and water exposures — Nevada, October 2012

	Case-patients (n = 22)	Controls (n = 24)		
				
Exposure	%*	%*	Odds ratio	p-value
Inadvertent swallowing of muddy water while competing	89	30	19.4	<0.001
Consumption of potable drinking water provided by race organizers	100	100	2.6	0.48
Consumption of food provided by race organizers	93	74	4.9	0.16
Full body submersion in surface water	94	96	0.7	0.86
Exposure of eyes or mouth to surface water or mud	100	74	6.4	0.09

* Denominator values varied as a result of nonreporting by some participants.
